# Cystoid Macular Edema 18 Years after Anterior Chamber Phakic Intraocular Lens Implantation

**DOI:** 10.1155/2022/1853248

**Published:** 2022-06-16

**Authors:** Elias L. Warrak, Majd S. Haddam, Walid N. Dandan, John E. Warrak, Fady K. Sammouh

**Affiliations:** ^1^Department of Ophthalmology and Visual Sciences, University of Balamand, Advanced Eye Care Hospital, Lebanon; ^2^University of Balamand, Faculty of Medicine and Health Sciences, Lebanon

## Abstract

We report a case of a 54-year-old female patient who underwent PAC-IOL implantation 18 years prior to presentation. The patient had a best corrected visual acuity (BCVA) 20/20 in the right eye (OD) postoperatively with normal eye exam on routine follow-up since then. The patient presented for acute onset decreased visual acuity in the right eye. BCVA was 20/60, and exam showed blunted macular reflex with no evidence of inflammation. Optical coherence tomography (OCT) showed CME. She was started on topical treatment (ketorolac 0.5%) OD four times daily. Three weeks later, the patient had a BCVA of 20/20 OD with a normal macular reflex and an OCT showing the resolution of the CME. To our knowledge, this is the first reported case of a CME 18 years post PAC-IOL implantation. The possible cause of this incidence could be related to subclinical intraocular inflammation. Ophthalmologists should be aware of the possibility of such a latent CME post-PAC-IOL implantation.

## 1. Introduction

The introduction of phakic anterior chamber IOL (PAC-IOL) by Strampelli and Barraquer in the 1950s has offered an innovative method to treat ametropia with a better and more stable visual acuity and a rapid recovery [1, 2]. The surgery was temporarily abandoned due to high rates of intraocular complications that were overcome in the 1980s with the introduction of new sites of implantation by Fechner and Baikoff [3–5]. The three known sites of phakic IOL implantation include posterior chamber IOL, anterior chamber angle fixated, and anterior chamber iris fixated [3]. The angle-supported PAC-IOL was introduced by Jan Worst in the 1980s with the phakic 6H® IOL introduced around 1998. Cystoid macular edema (CME) post-IOL implantation is one of the common complications causing eyesight deterioration usually within 3 to 12 weeks postoperatively due to vascular retinal disease and inflammatory cascades that particularly targets the macula [6]. The underlying mechanism is based on fluid accumulation in cyst-like spaces located within the neurosensory layers of the retina, mostly involving the outer plexiform layer, the watershed area of the retina [7]. This paper demonstrates a case of latent CME 18 years after the implantation of a PAC-IOL with complete resolution upon topical treatment.

## 2. Case Presentation

This is the case of a 54-year-old female patient with no prior medical history who presented to our facility with acute onset of blurred vision in her right eye of one-week duration 18 years after anterior chamber phakic intraocular lens (Phakic 6H®) placement. History goes back to November 2003 when the patient presented to our facility with interest in refractive surgery. She had an unremarkable ophthalmological history at that time. The best corrected visual acuity (BCVA) was 20/25 in the right eye (OD) and 20/20 in the left eye (OS) with a manifest refraction of −11.25 + 1.25 × 130° and −9.25 + 2.25 × 110°, respectively. Slit lamp examination and dilated funduscopy were unremarkable. Intraocular pressure using Goldmann applanation tonometry was 15 mmHg OD and 16 mmHg OS. Corneal topography showed a central corneal thickness of 504 *μ*m OD and 510 *μ*m OS. The highest keratometry (*K*_max_) values were 44.62 D OD and 45.58 D OS. The anterior chamber depth (ACD) was 3.42 mm OD and 3.25 mm OS. Central endothelial cell density by specular microscopy was 2645 cells/mm^2^ OD. Accordingly, laser-assisted in situ keratomileusis was performed for the left eye which yielded an uncorrected visual acuity of 20/20 measured two months after the surgery and PAC-IOL implantation was performed for the right eye. The risks and benefits of the procedure were explained to the patient prior to the procedure. PAC-IOL was implanted through a 6.25 mm superior corneal incision, followed by a surgical peripheral iridectomy (PI)superiorly. The nonfoldable Phakic 6H® lens was introduced into the anterior chamber and implanted at 6–12 o'clock orientation, with careful attention to proximity to the crystalline lens, iris, and endothelium. The wound was sutured with 10-0 nylon after verification of an open angle, and suture knots were buried. Antibiotic and steroid eye drops were given for four weeks postoperatively. Uncorrected visual acuity (UCVA) in the right eye was 20/30 at one and six months postoperatively. BCVA was 20/20 OD with a manifest refraction of −0.75 + 0.50 × 125 at the one and six months postoperative follow-ups. The patient was then routinely followed up with no complications and stable vision.

**Figure 1:**
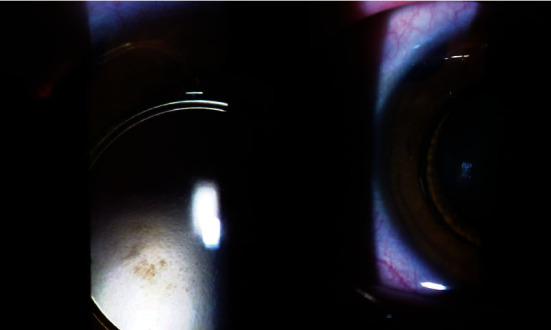
Slit lamp photo showing PAC-IOL with superior haptic migration into the PI.

**Figure 2:**
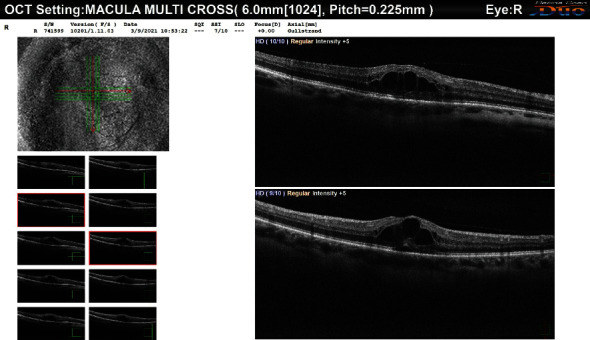
OCT scan showing CME 18 years after PAC-IOL implantation.

**Figure 3:**
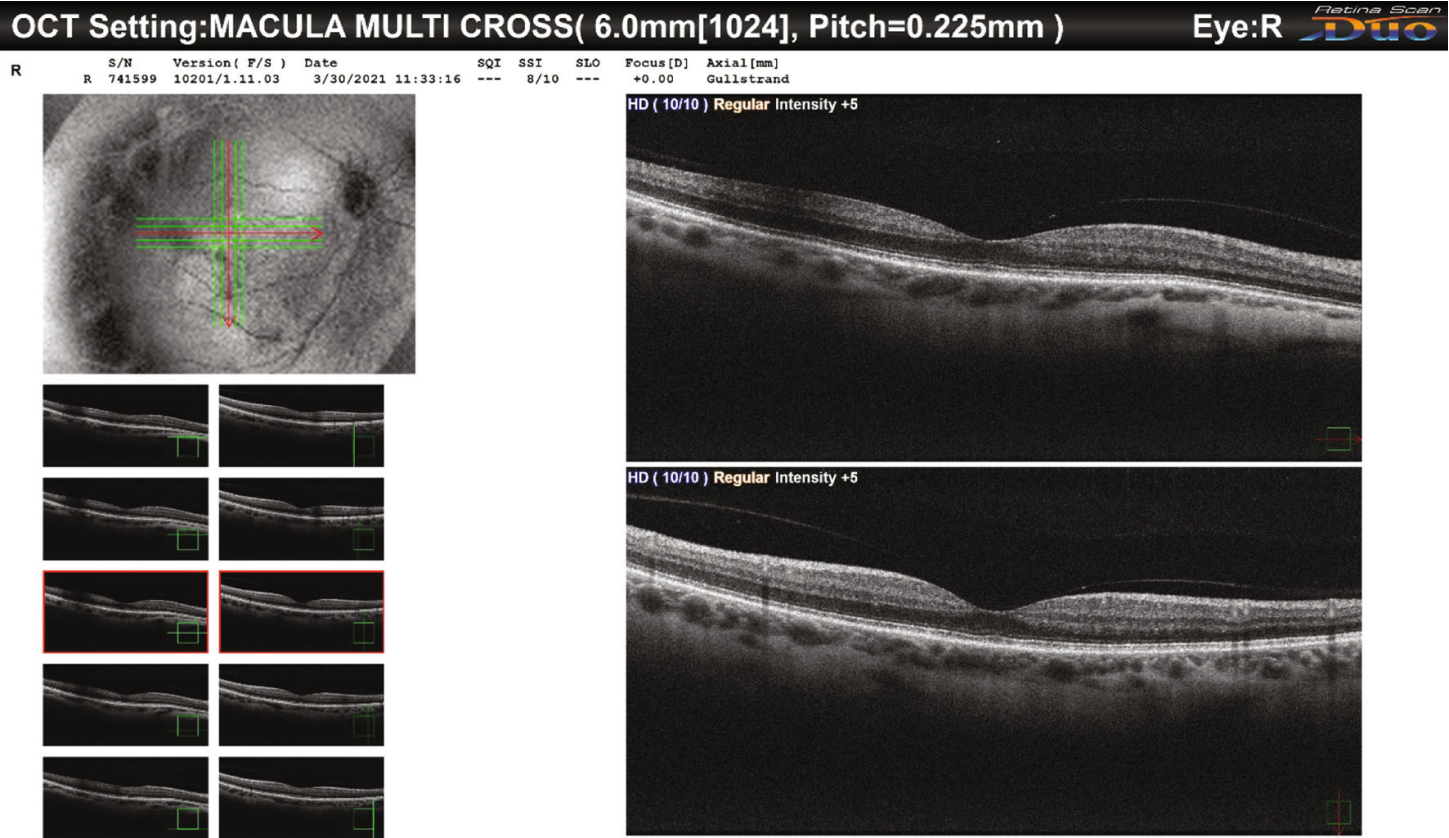
OCT scan showing resolution of CME 3 weeks after topical NSAID treatment.

On the day of presentation (March 2021), the patient complained of decreased visual acuity OD. BCVA was found to be 20/60 OD and 20/25 OS. Slit lamp examination for the right eye showed a deep and quiet anterior chamber, a clear cornea, an intact iris without any transillumination defects or synechia, and the PAC-IOL haptic migrated into the PI in the right eye (Figure 1). The sclera was white with no evidence of inflammatory changes. Dilated funduscopy showed clear vitreous, normal optic nerve, and retinal vasculature in both eyes. However, a blunted macular reflex with minimal macular edema was noted OD. Consequently, optical coherence tomography (OCT) was performed showing cystoid macular edema (CME) (Figure 2). The patient was started on topical nonsteroidal anti-inflammatory (NSAID) eye drops (ketorolac 0.5%) OD four times per day. Three weeks later, the BCVA improved to 20/20. The dilated fundus exam and repeat OCT showed the restoration of normal foveal anatomy and complete resolution of the CME in the right (Figure 3). Follow-up examination in one and three months revealed a normal slit lamp examination and funduscopy with a BCVA of 20/20 OD and 20/25 OS.

## 3. Discussion

Postsurgical cystoid macular edema (Irvine-Gass syndrome) is a major cause of reduced visual acuity secondary to phacoemulsification and other ocular surgeries and pathologies. It commonly occurs after penetrating keratoplasty, vitreoretinal procedures, IOL implantation, diabetes, uveitis, retinitis pigmentosa, and vascular occlusion [8]. Although the etiology is not fully clear, it is suggested that it is mainly mediated by postoperative inflammatory cascades and mediators that promote fluid accumulation in the perifoveal retina secondary to increased permeability of the perifoveal capillaries with possible direct macular traction [9]. In our case, the patient did not have any ocular surgeries or interventions except for the PAC-IOL implantation. Her medical history is negative for any systematic or autoimmune disease. Her biannual routine eye exam and funduscopy were always normal with no evidence of any inflammation.

Our patient selection criteria for PAC-IOL implantation procedure included a stable refractive error for more than 6 months, an anterior chamber depth greater than 3.0 mm, an endothelial cell count greater than 2500 cells/mm^2^, contact lens intolerance, BCVA of 20/200 or better, no evidence of cataract formation, and a corneal thickness not suitable for laser refractive surgery. Our patient was a good candidate for the Phakic 6H® implantation with all criteria applicable.

To our knowledge, this is the first report of a latent CME, 18 years after an ocular procedure. A previous case report demonstrated a refractory CME after toric ICL placement. The CME resolved 3 months after the explantation of the ICL with topical NSAID (Nepafenac®) treatment [10]. A case series involved six eyes of five patients who had iris fixated intraocular lens who suffered from chronic CME resistant to medical therapy. The visual acuity was severely impaired until the iris fixated intraocular lens was replaced by a scleral sutured IOL (with posterior vitrectomy for some cases) which led to complete resolution of the CME with improved visual acuity reaching 20/20 [11].

In our patient, a possible explanation for the latent CME that resolved completely by topical NSAIDs is that it occurred as a sequel of subclinical low-grade inflammation/uveitis. This subclinical uveitis can be considered since no evidence of anterior chamber inflammatory changes such as cells, flares, or keratic precipitates were observed in any of the follow-up visits. The contributing factors for this subclinical inflammation could be explained by three possibilities. First, the IOL could have minute surface defects rubbing on the iris. Second, the stability of the angle-fixated IOL could be distorted due to haptic migration or gradual iris atrophy secondary to microscopic movement of the PAC-IOL against the iris. Third, the haptics could be triggering microscopic trauma to the peripheral iris with subsequent low-grade inflammation/uveitis. In a case series involving 35 eyes with PAC-IOL followed 8 months to 8.5 years, the incidence of haptic migration into the superior PI was 23% but without any alteration on the BCVA, intraocular structures, or evidence of inflammation [12].

In conclusion, ophthalmologists should be aware about such cases of latent CME with no evident ocular inflammation that might respond well on topical conservative treatment. There are different approaches to CME that include topical NSAIDS, topical corticosteroids, oral carbonic anhydrase inhibitors, periocular and intravitreal corticosteroid injections, anti-VEGF intravitreal injections, and surgery in refractory cases. However, further studies need to be conducted at a larger scale to confirm our findings and construct standardized treatment protocols.
